# Evaluating WHO-Recommended Interventions for Preterm Birth: A Mathematical Model of the Potential Reduction of Preterm Mortality in Sub-Saharan Africa

**DOI:** 10.9745/GHSP-D-18-00402

**Published:** 2019-06-24

**Authors:** Jennifer B. Griffin, Alan H. Jobe, Doris Rouse, Elizabeth M. McClure, Robert L. Goldenberg, Beena D. Kamath-Rayne

**Affiliations:** a RTI International, Durham, NC, USA.; b Department of Pediatrics, University of Cincinnati College of Medicine, Cincinnati, OH, USA.; c Perinatal Institute, Cincinnati Children's Hospital Medical Center, Cincinnati, OH, USA.; d Department of Obstetrics and Gynecology, Columbia University, New York, NY, USA.; e Global Child Health, Cincinnati Children's Hospital Medical Center, Cincinnati, OH, USA.

## Abstract

**Background:**

Preterm birth, a leading cause of neonatal mortality, has the highest burden in low-income countries. In 2015, the World Health Organization (WHO) published recommendations for interventions to improve preterm outcomes. Our analysis uses the Maternal and Neonatal Directed Assessment of Technology (MANDATE) model to evaluate the potential effects that WHO-recommended interventions could have had on preterm mortality in sub-Saharan Africa in 2015.

**Methods:**

We modeled preterm birth subconditions causing mortality (respiratory distress syndrome, intraventricular hemorrhage, necrotizing enterocolitis, sepsis, birth asphyxia, and low birth weight). For each subcondition, models were populated with estimates of WHO-recommended intervention prevalence, case fatality, coverage, and efficacy. Various scenarios modeled improved coverage of single and combined interventions compared with baseline.

**Results:**

In 2015, approximately 500,000 neonatal deaths due to preterm birth occurred in sub-Saharan Africa. Single interventions with the greatest impact on preterm mortality included oxygen/continuous positive airway pressure (44,000 lives saved), cord care (38,500 lives saved), and breastfeeding (30,200 lives saved). Combined with improved diagnosis/transfer to a hospital, the impact of interventions showed greater reductions in mortality (oxygen/continuous positive airway pressure, 134,100 lives saved; antibiotics, 28,600 lives saved). Combined interventions had the greatest impact. Together, hospital delivery with comprehensive care for respiratory distress syndrome saved 190,600 lives, and comprehensive thermal care, breastfeeding, and prevention/treatment for sepsis saved 94,400 lives.

**Conclusion:**

In 2015, WHO-recommended interventions could have saved the lives of nearly 300,000 infants born preterm in sub-Saharan Africa. Combined interventions are necessary to maximize impact. Mathematical models such as MANDATE can estimate effects on health outcomes to allow health officials to prioritize implementation strategies.

## Abstract (precis)

Using the Maternal and Neonatal Directed Assessment of Technology (MANDATE) model, we estimate that WHO-recommended interventions could have saved nearly 300,000 lives in 2015. Combined interventions had the greatest impact. MANDATE can allow health officials to prioritize implementation strategies.

## INTRODUCTION

Preterm birth is the largest cause of neonatal mortality worldwide, with best estimates of 15 million infants affected yearly. Rates appear to increase in countries as data systems improve.^1^^,^^2^ Complications of preterm birth result in significant risks for developmental disability in survivors and high costs for long-term complex health care needs.^1^ In 2012, the *Born Too Soon* report highlighted the problem by publishing country-specific rates of preterm birth and calling for implementation of simple interventions that decreased preterm birth complications in high-income countries prior to the influence of neonatal intensive care.^3^ A further complication is that the causes of preterm birth are multifactorial, and classification of a phenotype of preterm birth is imprecise because of heterogeneous clinical presentations and confounding factors such as maternal malnutrition and infections.^4^^–^^6^

With the understanding that innovative solutions are needed to decrease mortality from preterm birth, the World Health Organization (WHO) published recommendations in 2015 on interventions to improve quality of care and outcomes surrounding preterm birth.^7^ The report detailed both maternal and neonatal interventions administered during pregnancy, labor, delivery, or the early neonatal period with the best available evidence for improving the incidence and adverse outcomes of preterm birth.^7^

Given limited resources and the priorities of governmental agencies and national subgroups to implement the guidelines, our aim was to identify interventions with greater effects on improving mortality due to preterm birth. Such interventions would be the focus of initial efforts at implementation. We used the Maternal and Neonatal Directed Assessment of Technology (MANDATE) model to evaluate WHO-recommended interventions for preterm birth to determine which interventions and/or bundle of interventions had the most impact in terms of lives saved.^8^

## METHODS

We considered the WHO-recommended interventions that could be provided during pregnancy, labor, and the neonatal period for reducing neonatal mortality in preterm infants.^7^ Recognizing that this list is not exhaustive in terms of additional challenges faced by premature infants, such as asphyxia and sepsis, we sought other literature to determine the WHO-recommended interventions for these conditions as well.^9^^–^^11^ Because WHO did not focus on interventions associated with the prevention or reduction of preterm birth (e.g., progestational agents), these were outside the scope of this analysis.

We used MANDATE, a nonstochastic, decision-tree model, to evaluate how WHO-recommended interventions would have influenced mortality in preterm neonates in sub-Saharan Africa in 2015. The methods used to develop the MANDATE model have been previously described.^8^^,^^12^ Briefly, we conducted a systematic review to populate variables regarding penetration, utilization, and efficacy of preventive, diagnostic, and treatment interventions specific to preterm birth in sub-Saharan Africa. Penetration, defined as the availability of an intervention, and utilization, defined as the appropriate use of an intervention, were considered in home, clinic, and hospital settings. Efficacy, defined as the ideal therapeutic effect of a given intervention, was treated as constant regardless of setting. MANDATE differentiates between the efficacy of diagnostics, which typically falls into 3 categories: (1) symptom recognition, made by a caregiver or unskilled care provider, frequently in a home setting; (2) clinical diagnostics, made by a skilled provider; and (3) technology-based diagnostics used to formally diagnose a condition.

We used MANDATE to evaluate how WHO-recommended interventions would have influenced mortality in preterm neonates.

We conducted the review, using PubMed, MEDLINE, the Cochrane Library, and WHO databases from 1980 to 2015 and the search terms “preterm,” “mort* OR death,” with “intervent* OR prevent* OR diagno* OR treat*” and “developing countries OR low income countries OR sub-Saharan Africa.” For intervention efficacy parameters, we used a modified GRADE system to prioritize higher quality data. Demographic and Health Surveys, United Nations, and WHO data were used to populate key parameters regarding the number of births, prevalence of prematurity, and case fatality rate data. Parameters were reviewed by experts on preterm mortality in low-income countries and were incorporated into the model. Model building was an iterative process, with calibration against high-level WHO estimates. The version of MANDATE used for this analysis can be accessed at http://mnhtech.org.

Table 1 summarizes the interventions recommended by WHO related to preterm birth mortality, as well as MANDATE model assumptions regarding the penetration, utilization, and efficacy of these interventions in sub-Saharan Africa.^7^^,^^9^^–^^11^ WHO recommendations included quantification of the strength of recommendations (weak, strong, or conditional) by considering the quality of evidence (graded as very low, low, moderate, or high) and were up to date as of 2015, but they are expected to be updated as new data accrue.^7^ MANDATE assumes births occur across different settings, including the home (50%), clinics (35%), and hospitals (15%), based on the most recently available Demographic and Health Survey data for sub-Saharan African countries. Additionally, we assumed that chlorhexidine use would be appropriate for all home births across sub-Saharan Africa, based on the uncertainty bounds for UNICEF-estimated neonatal mortality rates for sub-Saharan Africa in 2015. These generalizing assumptions may not reflect regional, country, and local variation in birth rates and neonatal mortality rates. The online model allows MANDATE users to change these assumptions to reflect additional data that may be available for a specific region or country, or to reflect other differences (e.g., urban/rural).

**TABLE 1.** WHO Interventions and Recommendations to Improve Preterm Birth Mortality, With MANDATE Model Assumptions of Intervention Penetration, Utilization, and Efficacy in Sub-Saharan Africa, 2015

Intervention	Recommendation Summary	WHO Strength of Recommendation for Implementation	Quality of Evidence	Baseline Penetration in MANDATE Home/Clinic/Hospital, %	Baseline Utilization in MANDATE Home/Clinic/Hospital, %	Efficacy in MANDATE Model, %	Key References
**Prenatal interventions for preterm**							
Antenatal corticosteroids	For women at risk of preterm birth (24–34 weeks gestation) under specific conditions	Strong	Moderate	0/10/50	0/5/25	RDS: 50 IVH: 42 NEC: 54	16–18,28
Antibiotics for preterm labor	For women with preterm prelabor rupture of membranes	Strong	Moderate	Not included in model			
**Postnatal care**							
Cord care	Daily CHX application to the umbilicus for newborns born at home in settings with high neonatal mortality. Clean, dry cord care for newborns born in health facilities and at home in low neonatal mortality settings.	Strong	Moderate	0/0/0	0/0/0	55	29–32
**Care of the preterm/LBW neonate**							
Thermal care for preterm newborns	KMC for the routine care of newborns weighing ≤2,000 g at birth, and should be initiated in health care facilities as soon as the newborns are clinically stable.	Strong	Moderate	95/95/95	0/0/2	51	15,33
	Unstable newborns weighing ≤2,000 g or stable newborns weighing ≤2,000 g who cannot be given KMC should be cared for in a thermo-neutral environment either under radiant warmers or in incubators.	Strong	Very low	0/0/50	0/0/30	60	34–36
Feeding	LBW infants, including those with very low birth weight, should be fed mother's own milk.	Strong	Moderate	99/99/99	20/40/55	Sepsis: 55 LBW: 18	37–39
**Management: newborn resuscitation**							
Immediate drying and additional stimulation	Newly born babies who do not breathe spontaneously after thorough drying should be stimulated by rubbing the back 2–3 times before cord clamping and PPV initiation.	Weak	Not graded	50/85/90	50/70/85	15	40–42
PPV	In newly born term or preterm (>32 weeks of gestation) babies requiring PPV, ventilation should be initiated with air.	Strong	Moderate	5/50/95	20/40/60	40	42–45
Oxygen therapy for preterm newborns	Ventilation of preterm babies born at or before 32 weeks of gestation with oxygen therapy with 30% oxygen or air (if blended oxygen is not available).	Strong	Very low	0/15/60	0/50/75	RDS: 25 Asphyxia: 25	46,47
**Management: RDS**							
Continuous positive airway pressure for newborns with RDS	Continuous positive airway pressure therapy is recommended for the treatment of preterm newborns with RDS.	Strong	Low	0/2/20	0/50/70	RDS: 50 Asphyxia: 50	46,47
Surfactant administration for newborns with RDS	Surfactant replacement therapy is recommended for intubated and ventilated newborns with RDS.	Conditional (health care facilities only with intubation, ventilator care, blood gas analysis, newborn nursing care and monitoring)	Moderate	0/1/5	0/50/75	35	46,48
**Management: neonatal sepsis**							
Prophylactic antibiotics for prevention of sepsis	A neonate with risk factors for infection (i.e., membranes ruptured > 18 hours before delivery, maternal fever > 38°C before delivery or during labor, or foul-smelling or purulent amniotic fluid) should be treated with the prophylactic antibiotics ampicillin and gentamicin for at least 2 days and reassessed if signs of sepsis or positive blood culture.	Weak	Very low	Not modeled			
Empirical antibiotics for suspected neonatal sepsis	Neonates with signs of sepsis should be treated with antibiotic treatment for at least 10 days.	Strong	Low	10/85/95	20/65/75	72	49,50
**Management: NEC**							
Antibiotics for treatment of NEC	Young neonates with suspected NEC should be treated with intravenous or intramuscular ampicillin (or penicillin) and gentamicin as first-line antibiotic treatment for 10 days.	Strong	Low	Not modeled			

Abbreviations: CHX, chlorhexidine; KMC, kangaroo mother care; IVH, intraventricular hemorrhage; LBW, low birth weight; MANDATE, Maternal and Neonatal Directed Assessment of Technology; NEC, necrotizing enterocolitis; PPV, positive pressure ventilation; RDS, respiratory distress syndrome; WHO, World Health Organization.

For each subcondition affecting preterm mortality, the model was populated with the estimated prevalence, the case fatality rate, and WHO-recommended interventions to prevent preterm mortality (Table 2). Subconditions included direct causes of preterm mortality, including respiratory distress syndrome (RDS), intraventricular hemorrhage (IVH), and necrotizing enterocolitis (NEC), sepsis, birth asphyxia, and low birth weight. Each scenario models 2 levels of improvement: (1) an incremental care model, in which penetration and utilization are increased by 20% from current care estimates, with maximum penetration and utilization set at 98%; and (2) a universal coverage model, in which penetration and utilization are set to 98% for each intervention.

**TABLE 2.** Additional MANDATE Model Assumptions, Sub-Saharan Africa, 2015

Assumptions	Value
**Preterm births in sub-Saharan Africa, N**	3,988,000
**Delivery location in sub-Saharan Africa, %**	
Home	50
Clinic	35
Hospital	15
**Antenatal care location in sub-Saharan Africa, %**	
Home	30
Clinic	65
Hospital	5
**Preterm subconditions contributing to preterm mortality, Prevalence | Case fatality rate,^a^ %**	
Respiratory distress	20 | 35
Intraventricular hemorrhage	7 | 7.5
Necrotizing enterocolitis	1 | 25
Sepsis	9 | 40
Birth asphyxia	20 | 20
Preterm with no other conditions	43 | 2.1
**Diagnostics, Baseline penetration | Baseline utilization | Efficacy for Home/Clinic/Hospital, %**	
Preterm labor^51^^–^^53^	50/85/90 | 5/20/35 | 25/80/80
Respiratory distress syndrome^46^^,^^54^	50/85/90 | 40/60/95 | 75/95/95
Intraventricular hemorrhage^54^	50/85/90 | 5/40/70 | 25/45/45
Necrotizing enterocolitis^54^^–^^56^	50/85/90 | 5/40/70 | 25/85/85
Sepsis^49^^,^^57^^,^^58^	95/85/90 | 75/80/90 | 75/95/95
Low birth weight^59^	50/85/90 | 5/75/90 | 25/95/95

Abbreviation: MANDATE, Maternal and Neonatal Directed Assessment of Technology.

a The prevalence and case fatality rates assume no preventive or treatment interventions.

Baseline MANDATE estimates of the number of neonatal preterm deaths associated with subconditions impacting preterm mortality in sub-Saharan Africa in 2015 can be seen in the Figure. The methods describing the calculation of baseline mortality estimates have been previously published.^12^ In short, we began with sub-Saharan African pregnancies in 2015; calibrated the model using historic rates for the various conditions (Table 2); estimated the impact of interventions using penetration, utilization, and efficacy (Table 1); and applied untreated case fatality rates (Table 2) in order to estimate baseline mortality. Direct complications of preterm birth contributing to preterm mortality, including RDS, IVH, and NEC, were estimated to cause 303,400 deaths, consistent with other reports of preterm deaths due to direct complications in sub-Saharan Africa.^13^^,^^14^ Other subconditions associated with preterm mortality included sepsis and birth asphyxia. Finally, prematurity alone posed an increased risk of death among preterm neonates. These preterm babies at risk of mortality were captured in the model subcondition “low birth weight.” MANDATE estimated approximately 500,000 total preterm deaths in 2015 associated with direct and indirect conditions that contribute to preterm mortality in sub-Saharan Africa. We modeled the impact of WHO-recommended interventions for each subcondition potentially causing preterm death and summarized scenarios for each subcondition and associated interventions impacting preterm mortality. All scenarios are compared with the baseline, current-care scenario. In most cases, results are rounded to the nearest 100 preterm deaths.

**FIGURE:**
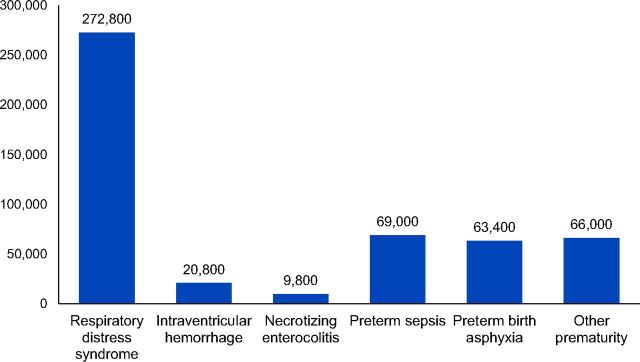
MANDATE Model Estimates of the Number of Preterm Deaths Associated With Subconditions Impacting Preterm Mortality, Sub-Saharan Africa, 2015 Abbreviation: MANDATE, Maternal and Neonatal Directed Assessment of Technology.

MANDATE estimated approximately 500,000 deaths in 2015 associated with conditions that contribute to preterm mortality.

## RESULTS

In Table 3, we report the number of deaths from preterm-associated mortality from RDS, IVH, and NEC in sub-Saharan Africa in 2015. With interventions at current levels of use, approximately 300,000 preterm deaths can be attributed to RDS, IVH, and NEC (scenario 1). In the first set of WHO-recommended interventions, even in the universal coverage models, only small to moderate impacts on preterm mortality were present, with 300 lives saved with improved surfactant use in hospitals (scenario 2), 5,000 lives saved in the antenatal corticosteroid model (scenario 3), and 42,300 lives saved in the improved oxygen/continuous positive airway pressure (CPAP) model (scenario 4). Thus, there was a decrease in mortality of approximately 14%. The second set of scenarios evaluated improved diagnosis with and without transfer. Improved diagnosis of preterm labor alone had a smaller impact (scenario 5) compared with diagnosis with transfer to a higher-level facility (scenario 6), with 2,100 lives saved compared with baseline in the incremental change model and 16,300 lives saved in the universal coverage model. Combining improved diagnostics and transfer to a higher-level care with single interventions (scenarios 7 and 8) demonstrated the synergistic effects of improving diagnostics, transfers, and treatments. For example, improved diagnosis of RDS paired with transfer and improved CPAP prevented 16,000 preterm deaths in the incremental change model and 127,300 deaths in the universal coverage model, a 42% reduction. In the final set of scenarios, we show that incremental and near universal improvements in diagnosis and transfer with WHO-packaged interventions would have the greatest impact on preterm mortality. For example, improved preterm labor diagnosis, transfer, antenatal corticosteroids, surfactant, and oxygen/CPAP jointly prevented approximately 112,000 preterm deaths (scenario 10) and improved diagnosis of respiratory distress, transfer, surfactants in hospitals, and oxygen/CPAP have prevented 155,700 preterm deaths in the universal coverage model, a reduction of nearly half (scenario 11). In the model with the greatest impact, scenario 12, all preterm deliveries were assumed to occur in hospital settings, with improved antenatal corticosteroid use and diagnosis and treatment of respiratory distress, including surfactants and oxygen/CPAP, thereby preventing the deaths of 190,600 preterm infants in this universal coverage model, a mortality reduction of nearly two-thirds.

**TABLE 3.** Impact of ANCS and Other WHO-Recommended Interventions^a^ to Prevent Preterm Mortality From RDS, IVH, and NEC, Sub-Saharan Africa, 2015

Scenario No.	Scenario	Incremental Change Model^b^		Universal Coverage Model^c^	
		Preterm Deaths, No.^d^	Preterm Deaths Prevented Compared With Current Level of Care, No. (%)	Preterm Deaths, No.	Preterm Deaths Prevented Compared With Current Level of Care, No. (%)
1	Current levels of prevention, diagnosis, and treatment	303,400	N/A	303,400	N/A
**Improved WHO single interventions**					
2	Increased surfactant in hospital settings for RDS	303,300	100 (<0.1)	303,100	300 (0.1)
3	Increased ANCS in hospital settings for RDS, IVH, and NEC	302,300	1,100 (0.4)	298,400	5,000 (1.7)
4	Increased oxygen/CPAP in hospital and clinical settings for RDS	295,300	8,100 (2.7)	261,100	42,300 (13.9)
**Improved diagnosis of preterm labor and transfer with current care**					
5	Increased diagnosis of preterm labor birth, with current levels of care for RDS, IVH, and NEC	302,400	1,000 (0.3)	299,900	3,500 (1.2)
6	Increased diagnosis of imminent preterm birth and transfer to hospitals, with current levels of care for RDS, IVH, and NEC	301,300	2,100 (0.7)	287,100	16,300 (5.4)
**Improved diagnosis and transfer with WHO single interventions**					
7	Increased diagnosis of respiratory distress, transfer, and surfactant (hospitals only) for RDS	299,900	3,500 (1.2)	282,800	20,600 (6.8)
8	Increased diagnosis of imminent preterm birth, transfer to hospitals, and ANCS (hospitals only) for RDS, IVH, and NEC	298,600	4,800 (1.6)	236,700	66,700 (22.0)
9	Increased diagnosis of respiratory distress, transfer, and oxygen/CPAP for preterm RDS	287,400	16,000 (5.3)	176,100	127,300 (42.0)
**Improved diagnosis and transfer with WHO packaged interventions**					
10	Improved diagnosis of imminent preterm birth, transfer to hospitals, ANCS (hospitals only), and treatment with surfactants (hospitals only) and oxygen/CPAP for RDS, IVH, and NEC	289,700	13,700 (4.5)	191,300	112,100 (37.0)
11	Increased diagnosis of respiratory distress, transfer to hospitals, and treatment, including surfactants (hospitals only) and oxygen/CPAP for RDS	286,900	16,500 (5.4)	155,700	147,711 (48.7)
12	Hospital delivery for all preterm birth, with ANCS (hospitals only), improved diagnosis and treatment of respiratory distress, including surfactants (hospitals only) and CPAP for RDS, IVH, and NEC	223,300	80,100 (26.4)	112,800	190,600(62.8)

Abbreviations: ANCS, antenatal corticosteroids; CPAP, continuous positive airway pressure; IVH, intraventricular hemorrhage; NEC, necrotizing enterocolitis; RDS, respiratory distress syndrome; WHO, World Health Organization.

a Assumptions regarding baseline penetration and utilization of interventions including ANCS, surfactant, and CPAP as shown in Table 1. Assumptions regarding diagnostics and transfers found in Table 2.

b The incremental change model assumes 20% increase from baseline penetration and utilization.

c The universal coverage model assumes 98% penetration and utilization of interventions.

d All estimates rounded to nearest 100.

In the model with the greatest impact, 190,600 deaths due to RDS, IVH, and NEC were prevented.

In Table 4, we report the number of deaths from preterm-associated mortality from sepsis, birth asphyxia, and low birth weight in sub-Saharan Africa in 2015. We estimated preterm mortality associated with sepsis, birth asphyxia, and low birth weight with current levels of care, including low to moderate coverage of positive pressure ventilation (PPV), oxygen, cord care, breastfeeding, and antibiotics, to be approximately 198,000 deaths (scenario 1). In the first set of improved WHO single intervention scenarios, we found small to moderate impacts on preterm mortality, with near-universal oxygen/CPAP for birth asphyxia saving 1,700 lives (scenario 2); PPV for birth asphyxia saving 4,200 lives (scenario 3); drying and stimulation of newborns saving 3,000 lives (scenario 4); thermal care, including kangaroo mother care (KMC), saving 9,100 lives (scenario 5); antibiotics saving 18,200 lives (scenario 6); breastfeeding, reducing mortality from both sepsis and LBW, saving 30,200 lives (scenario 7); and chlorhexidine in home settings and dry cord care in clinical settings saving 38,500 lives, a reduction of nearly 20% (scenario 8). The second set of scenarios shows that improved diagnosis and transfer on preterm mortality from sepsis, birth asphyxia, and low birth weight had relatively small impacts on preterm mortality, with a range from diagnosis of birth asphyxia saving 1,900 lives (scenario 9) to sepsis diagnosis with transfer to clinical settings saving 14,300 lives (scenario 13). The third set of scenarios demonstrates the increased impact of improved diagnosis and transfer with WHO-recommended single interventions, with diagnosis and transfer for birth asphyxia with oxygen support saving 6,800 lives (scenario 13); diagnosis of birth asphyxia with transfer and PPV saving 8,600 lives (scenario 14); and improved sepsis diagnosis and transfer with antibiotics saving 28,600 lives (scenario 15). In the last set of scenarios, combined interventions had the greatest impact on preterm mortality. Impacts ranged from 26,200 lives saved with comprehensive care for birth asphyxia (scenario 16); to 59,100 lives saved with comprehensive prevention and treatment of sepsis; to 94,400 lives saved with thermal care and breastfeeding (scenario 17), in addition to comprehensive care for birth asphyxia and sepsis (scenario 18).

**TABLE 4.** Impact of WHO-Recommended Interventions^a^ to Prevent Preterm Mortality From Sepsis, Birth Asphyxia, and Low Birth Weight, Sub-Saharan Africa, 2015

Scenario No.	Scenario	Incremental Change Model^b^		Universal Coverage Model^c^	
		Preterm Deaths, No.^d^	Preterm Deaths Prevented Compared With Current Level of Care, No. (%)	Preterm Deaths, No.	Preterm Deaths Prevented Compared With Current Level of Care, No. (%)
1	Current levels of prevention, diagnosis, and treatment	198,400	N/A	198,400	N/A
**Improved WHO single interventions**					
2	Oxygen/CPAP for birth asphyxia in clinics and hospitals	198,000	400 (0.2)	196,800	1,700 (0.9)
3	PPV for birth asphyxia in all settings	197,200	1,200 (0.6)	195,100	4,200 (2.1)
4	Drying and stimulation for birth asphyxia in all settings	196,486	1,900 (1.0)	195,400	3,000 (1.5)
5	Thermal care for LBW, including KMC in all settings and warmers in hospital settings	196,000	2,500 (1.3)	189,400	9,100 (4.6)
6	Antibiotics for suspected neonatal sepsis in all settings	192,100	6,300 (3.2)	180,300	18,200 (9.1)
7	Breastfeeding for sepsis and LBW in all settings	189,300	9,100 (4.6)	168,200	30,200 (15.2)
8	Chlorhexidine for sepsis in home settings and dry cord care in clinical settings	190,800	7,600 (3.8)	159,900	38,500 (19.4)
**Improved diagnosis and transfer with current care**					
9	Diagnosis of birth asphyxia and need for postresuscitation care, with current levels of care	197,200	1,300 (0.7)	196,500	1,900 (1.0)
10	Diagnosis of birth asphyxia and need for postresuscitation care and improved transfer to hospitals, with current levels of care	197,000	1,400 (0.7)	196,200	2,200 (1.1)
11	Diagnosis of sepsis, with current levels of care	194,700	3,700 (1.9)	194,300	4,200 (2.1)
12	Diagnosis of sepsis and transfer to hospitals, with current levels of care	187,400	11,000 (5.5)	184,100	14,300 (7.2)
**Improved diagnosis and transfer with WHO single treatment interventions**					
13	Diagnosis of birth asphyxia and need for postresuscitation care, transfer, and oxygen/CPAP	196,300	2,100 (1.1)	191,700	6,800 (3.4)
14	Diagnosis of birth asphyxia and need for postresuscitation care, transfer, and positive pressure ventilation	195,500	2,900 (1.5)	189,800	8,600 (4.3)
15	Diagnosis of sepsis, transfer, and antibiotics for suspected neonatal sepsis	180,800	17,600 (8.9)	169,800	28,600 (14.3)
**Improved diagnosis and transfer with WHO-packaged interventions**					
16	Drying and stimulation, diagnosis of birth asphyxia and need for postresuscitation care, transfer to hospitals, and treatment, including PPV and oxygen/CPAP	188,057	10,400 (5.2)	172,200	26,200 (13.2)
17	Cord care and breastfeeding, diagnosis of sepsis, transfer, and antibiotics for suspected neonatal sepsis	169,200	29,200 (14.7)	139,400	59,100 (29.8)
18	Packaged interventions 16 and 17, with increased thermal care and breastfeeding for LBW	159,300	39,100 (19.7)	104,000	94,400 (47.6)

Abbreviations: CPAP, continuous positive airway pressure; KMC, kangaroo mother care; LBW, low birth weight; PPV, positive pressure ventilation; WHO, World Health Organization.

a Assumptions regarding baseline penetration and utilization of interventions including ANCS, surfactant, and CPAP as shown in Table 1. Assumptions regarding diagnostics and transfers found in Table 2.

b The incremental change model assumes 20% increase from baseline penetration and utilization.

c The universal coverage model assumes 98% penetration and utilization of interventions.

d All estimates rounded to nearest 100.

Combined interventions had the greatest impact on prevention of preterm mortality.

## DISCUSSION

To improve neonatal mortality worldwide, the burden of preterm birth-related deaths must be lessened. The *Born Too Soon* report sets a target of 50% reduction in preterm deaths in countries with a neonatal mortality rate above 5 per 1,000 live births by 2025. Toward this end, closing the gap on the higher incidence mortality from preterm birth and its subsequent complications, specifically in low- and middle-income countries, is one of the top priorities.^3^ To aid policy makers and frontline health providers, this study showcases the single interventions or bundles of interventions recommended by WHO that could potentially have the greatest effect on reducing preterm birth mortality.

The single interventions with the greatest impact on preterm mortality are oxygen/CPAP, cord care, breastfeeding, and antibiotics. Interestingly, in a Lives Saved Tool (LiST) analysis of preterm birth interventions, including family planning, antenatal corticosteroids, antibiotics for prolonged premature rupture of membranes, immediate assessment and simple care of all babies, neonatal resuscitation, thermal care, and KMC, 84% of premature babies could be saved if these interventions were made universally available (95%).^14^ In that study, the 2 single interventions that had the greatest impact on preterm birth mortality were antenatal corticosteroids and KMC.^14^^–^^16^ The terms of our analysis were different. We modeled lives saved for sub-Saharan Africa for a single year (2015), while the LiST analysis considered lives saved for 2 separate periods of time (2010–2015) and then through 2025. Furthermore, the LiST analysis did not include the single intervention that had the greatest effect in our analysis: oxygen/CPAP for RDS. Our model assumptions were also different; MANDATE assumes that treatments are relevant only to preterm neonates with/without particular subconditions. For example, we assumed that KMC will only be efficacious in stable preterm neonates without critical subconditions needing treatment, such as RDS or sepsis, while LiST assumes that KMC is relevant to the entire population of preterm neonates. We also assumed that receipt of treatment interventions is dependent on a previous diagnosis, while LiST modeled the combined effect of multiple interventions. Finally, the MANDATE model provides the opportunity to evaluate the location where the intervention is implemented and account for transfer to higher levels of care; for example, it is unlikely that oxygen could be implemented in a home setting, but is likely to be used in a hospital setting.

Our modeling found that the single interventions with the greatest impact on preterm mortality are oxygen/CPAP, cord care, breastfeeding, and antibiotics.

The benefits of antenatal corticosteroids in low-resource settings are unclear. A recent WHO multicountry survey on maternal and newborn health indicated that current national coverage estimates varied, between 16% and 91%, with a median of 54%.^17^ A multicountry cluster-randomized trial done by the National Institute of Child Health and Human Development (NICHD) Global Network, the ACT trial, demonstrated increased 28-day neonatal mortality possibly explained by maternal infection in the corticosteroid-exposed group.^18^ Furthermore, in the ACT trial, only 16% of women who were given corticosteroids gave birth to an infant below the 5th percentile for weight, indicating unnecessary overexposure to the treatment.^18^^,^^19^ Secondary analysis of the data from the Guatemala site suggested that the combination of improved quality of obstetric and neonatal care in facilities associated with antenatal corticosteroid treatment may have reduced neonatal mortality.^20^ A randomized controlled trial of late preterm infants (34 weeks to 36 weeks, 5 days) by the NICHD Maternal Fetal Medicine Unit Network demonstrated modest improvement in neonatal respiratory morbidity in older preterm infants with antenatal corticosteroid exposure, but an increased risk of hypoglycemia.^21^ Given concerns about safety and efficacy of antenatal corticosteroids in low-resource settings, WHO has strict criteria for their use, including accurate assessment of gestational age, imminent preterm birth, no clinical evidence of maternal infection, adequate childbirth care (recognition and management of preterm labor and birth), and adequate care for preterm newborn (including resuscitation, thermal care, feeding support, infection treatment, and safe oxygen use).^7^ An international research collaboration called WHO ACTION (Antenatal Corticosteroids for Improving Outcomes in preterm Newborns) is conducting 2 concurrent placebo-controlled efficacy trials of antenatal corticosteroids (dexamethasone) that will eventually enroll over 28,000 women.^19^ For our analysis, we have assumed benefit only for antenatal corticosteroids based on the magnitude of effect in the multiple randomized controlled trials, primarily performed in high-income countries.

Combined interventions together with transfer to the hospital had the greatest impact on lives saved. This outcome is likely because hospitals have greater availability of some of the recommended interventions and are more likely to use an intervention, if available. However, the model assumes that interventions are being utilized at a gold standard level, which frequently will not be accurate. A study in India determined that while rates of institutional deliveries in South and Central India increased, and perinatal and stillbirth mortality decreased, neonatal mortality did not change.^22^ A 10-country analysis of skilled birth attendance demonstrated that less than 10% of mothers who saw a skilled birth attendant once during pregnancy received a set of 8 key interventions, and quality of care did not increase as number of antenatal visits increased.^23^^,^^24^ Furthermore, it is important to recognize that even when interventions are utilized, they may not change outcomes. For example, a recent cluster randomized study evaluated the use of the WHO Safe Childbirth Checklist accompanied by 8 months of coaching in facilities across India.^25^ While there was increased adherence to the 18 essential birth practices in the intervention group, maternal and perinatal mortality did not differ between groups.^25^ The study exemplifies the difficulty in achieving the high reliability of gold standard performance, even for the most evidence-based practices; for example, birth attendants performed hand hygiene in 35% cases in intervention groups.^25^ Clearly, complex relationships exist between quality of care and outcomes that are beyond the scope of what the MANDATE model, or in fact any model, can predict.^25^

Combined interventions together with transfer to the hospital had the greatest impact on lives saved.

Our study has several other limitations. The assumptions in the model are based upon our best efforts to find primary sources that document the effects of interventions in low- and middle-income countries; therefore, the list of interventions included in our study is not exhaustive. While MANDATE has the ability to look with more granularity at some of the subconditions and the locations where interventions may occur, the lack of primary source data to populate our assumptions is an identified gap, which underscores the need for greater documentation and research in these areas. Due to varied quality of primary sources from a range of countries across sub-Saharan Africa, MANDATE makes the simplifying assumption that baseline condition incidence and intervention penetration and utilization are the same across the sub-Saharan Africa continent. MANDATE is a nonstochastic model decision tree model that does not model uncertainty. Furthermore, we could not distinguish between the effects of separate interventions for different degrees of prematurity (e.g., extremely preterm versus late preterm infants) or the interaction of multiple pathologies that may lead to a poor outcome. In some locales with extremely limited resources, we were also unable to determine if there was a threshold of prematurity below which interventions would be counter-productive because the chance of intact survival is so low. Finally, our study was not able to evaluate the possible adverse consequence of broader coverage of some interventions, for example, unnecessary treatment with antenatal corticosteroids or inappropriate overuse of oxygen in preterm infants, which could result in excess morbidity or mortality.

Moving forward, reducing preterm birth mortality requires improving coverage of evidence-based interventions that are known to reduce preterm birth-associated mortality,^26^^,^^27^ but then carefully quantifying the effects. The WHO guideline also described steps toward successful dissemination and implementation of the recommended interventions. However, the introduction of evidence-based policies to improve preterm birth outcomes depends on well-planned and participatory consensus-driven processes of adaptation and implementation.^7^ Evidence-based approaches to facilitate this knowledge and transfer exchange include collective impact collaboratives that bring together multiple sectors to achieve policy change through a common agenda, shared measurement systems, mutually reinforcing activities, continuous communication, and the presence of a backbone organization, as well as learning collaboratives that bring policy makers together in an ongoing way to share knowledge about a specific health outcome.^26^ To address issues of implementation and dissemination, further national and subnational groups will need support to adapt and implement the WHO-recommended interventions and change the beliefs and behaviors of local health care providers. The published recommendations specifically document anticipated barriers to implementation and possible steps to mitigate these challenges. Some of the anticipated barriers included nonavailability or an irregular supply of essential medicines, lack of human resources with expertise and skill to implement recommended practices and monitor response, low certainty of gestational age estimation in low-resource environments, and lack of effective referral mechanisms and care that ensure management of women with preterm labor and preterm infants occurs within a continuum of care.

Finally, while the WHO-recommended guidelines represent the best available evidence-based interventions to decrease preterm birth mortality, further research is urgently needed for preterm birth prevention. A recent study indicated that while the strongest individual risk factor of preterm birth is previous preterm birth and preeclampsia, more than 65% of the total aggregated risk of preterm birth lacked a plausible biologic explanation. In addition, 63% of the differences in prematurity rates between countries could not be explained with known factors.^2^ New efforts to better classify the characteristics of the preterm birth syndrome, its clinical phenotypes, and core outcomes for evaluation of interventions will aid in focusing and accelerating research on this complicated topic.

## Notes

**Peer Reviewed**

**Funding:** This research was funded by a grant from the Bill & Melinda Gates Foundation to RTI International. The Gates Foundation did not play a role in the collection, analysis, or presentation of these data.

## References

[1] Blencowe H Cousens S Chou D.; Born Too Soon Preterm Birth Action Group. Born too soon: the global epidemiology of 15 million preterm births. Reprod Health. 2013;10(suppl 1):S2. 10.1186/1742-4755-10-S1-S2. 24625129 PMC3828585

[2] Ferrero DM Larson J Jacobsson B. Cross-country individual participant analysis of 4.1 million singleton births in 5 countries with very high human development index confirms known associations but provides no biologic explanation for 2/3 of all preterm births. PLoS One. 2016;11(9):e0162506. 10.1371/journal.pone.0162506. 27622562 PMC5021369

[3] March of Dimes; Partnership for Maternal, Newborn and Child Health; Save the Children; World Health Organization (WHO). Born Too Soon: The Global Action Report on Preterm Birth. Geneva: WHO; 2012. http://www.who.int/pmnch/media/news/2012/201204_borntoosoon-report.pdf. Accessed April 23, 2019.

[4] Goldenberg RL Gravett MG Iams J. The preterm birth syndrome: issues to consider in creating a classification system. Am J Obstet Gynecol. 2012;206(2):113–118. 10.1016/j.ajog.2011.10.865. 22177186

[5] Kramer MS Papageorghiou A Culhane J. Challenges in defining and classifying the preterm birth syndrome. Am J Obstet Gynecol. 2012;206(2):108–112. 10.1016/j.ajog.2011.10.864. 22118964

[6] Villar J Papageorghiou AT Knight HE. The preterm birth syndrome: a prototype phenotypic classification. Am J Obstet Gynecol. 2012;206(2):119–123. 10.1016/j.ajog.2011.10.866. 22177191

[7] World Health Organization (WHO). WHO Recommendations on Interventions to Improve Preterm Birth Outcomes. Geneva: WHO; 2015. http://apps.who.int/iris/bitstream/10665/183037/1/9789241508988_eng.pdf. Accessed April 23, 2019. 26447264

[8] McClure EM Rouse DJ MacGuire ER. The MANDATE model for evaluating interventions to reduce postpartum hemorrhage. Int J Gynaecol Obstet. 2013;121(1):5–9. 10.1016/j.ijgo.2012.10.030. 23313144 PMC3628756

[9] World Health Organization (WHO). Guidelines on Basic Neonatal Resuscitation. Geneva: WHO; 2012. https://www.who.int/maternal_child_adolescent/documents/basic_newborn_resuscitation/en/. Accessed April 23, 2019.

[10] World Health Organization (WHO). Guideline: Managing Possible Serious Bacterial Infection in Young Infants When Referral Is Not Feasible. Geneva: WHO; 2015. https://www.who.int/maternal_child_adolescent/documents/bacterial-infection-infants/en/. Accessed April 23, 2019. 26447263

[11] World Health Organization (WHO). WHO Recommendations on Newborn Health: Guidelines Approved by the WHO Guidelines Review Committee. Geneva: WHO; 2017. https://www.who.int/maternal_child_adolescent/documents/newborn-health-recommendations/en/. Accessed April 23, 2019.

[12] Jones-Hepler B Moran K Griffin J. Maternal and Neonatal Directed Assessment of Technologies (MANDATE): methods and assumptions for a predictive model for maternal, fetal, and neonatal mortality interventions. Glob Health Sci Pract. 2017;5(4):571–580. 10.9745/GHSP-D-16-00174. 29284695 PMC5752604

[13] Lawn J Mongi P Cousens S. Africa's newborns–counting them and making them count. In: Opportunities for Africa's Newborns: Practical Data, Policy and Programmatic Support for Newborn Care in Africa. Partnership for Maternal, Newborn and Child Health; 2006:11–22. https://www.who.int/pmnch/media/publications/aonsection_I.pdf. Accessed April 23, 2019.

[14] Lawn JE Kinney MV Belizan JM.; Born Too Soon Preterm Birth Action Group. Born too soon: accelerating actions for prevention and care of 15 million newborns born too soon. Reprod Health. 2013;10(suppl 1):S6. 10.1186/1742-4755-10-S1-S6. 24625252 PMC3828574

[15] Lawn JE Mwansa-Kambafwile J Horta BL Barros FC Cousens S. ‘Kangaroo mother care’ to prevent neonatal deaths due to preterm birth complications. Int J Epidemiol. 2010;39(suppl 1):i144–i154. 10.1093/ije/dyq031. 20348117 PMC2845870

[16] Mwansa-Kambafwile J Cousens S Hansen T Lawn JE. Antenatal steroids in preterm labour for the prevention of neonatal deaths due to complications of preterm birth. Int J Epidemiol. 2010;39(suppl 1):i122–i133. 10.1093/ije/dyq029. 20348115 PMC2845868

[17] Vogel JP Souza JP Gülmezoglu AM.; WHO Multi-Country Survey on Maternal and Newborn Health Research Network. Use of antenatal corticosteroids and tocolytic drugs in preterm births in 29 countries: an analysis of the WHO Multicountry Survey on Maternal and Newborn Health. Lancet. 2014;384(9957):1869–1877. 10.1016/S0140-6736(14)60580-8. 25128271

[18] Althabe F Belizán JM McClure EM. A population-based, multifaceted strategy to implement antenatal corticosteroid treatment versus standard care for the reduction of neonatal mortality due to preterm birth in low-income and middle-income countries: the ACT cluster-randomised trial. Lancet. 2015;385(9968):629–639. 10.1016/S0140-6736(14)61651-2. 25458726 PMC4420619

[19] Vogel JP Oladapo OT Pileggi-Castro C. Antenatal corticosteroids for women at risk of imminent preterm birth in low-resource countries: the case for equipoise and the need for efficacy trials. BMJ Glob Health. 2017;2(3):e000398. 10.1136/bmjgh-2017-000398. 29082019 PMC5656119

[20] Garces A McClure EM Figueroa L. A multi-faceted intervention including antenatal corticosteroids to reduce neonatal mortality associated with preterm birth: a case study from the Guatemalan Western Highlands. Reprod Health. 2016;13(1):63. 10.1186/s12978-016-0178-0. 27221237 PMC4877983

[21] Gyamfi-Bannerman C Thom EA Blackwell SC.; NICHD Maternal–Fetal Medicine Units Network. Antenatal betamethasone for women at risk for late preterm delivery. N Engl J Med. 2016;374(14):1311–1320. 10.1056/NEJMoa1516783. 26842679 PMC4823164

[22] Goudar SS Goco N Somannavar MS. Institutional deliveries and perinatal and neonatal mortality in Southern and Central India. Reprod Health. 2015;12(suppl 2):S13. 10.1186/1742-4755-12-S2-S13. 26063586 PMC4464025

[23] UNICEF. Committing to Child Survival: A Promise Renewed. Progress Report 2014. New York: UNICEF; 2014. http://files.unicef.org/publications/files/APR_2014_web_15Sept14.pdf. Accessed April 23, 2019.

[24] Wardlaw T You D Hug L Amouzou A Newby H. UNICEF Report: enormous progress in child survival but greater focus on newborns urgently needed. Reprod Health. 2014;11(1):82. 10.1186/1742-4755-11-82. 25480451 PMC4320591

[25] Semrau KEA Hirschhorn LR Marx Delaney M.; BetterBirth Trial Group. Outcomes of a coaching-based WHO Safe Childbirth Checklist program in India. N Engl J Med. 2017;377(24):2313–2324. 10.1056/NEJMoa1701075. 29236628 PMC5672590

[26] Yamey G Horváth H Schmidt L Myers J Brindis CD. Reducing the global burden of preterm birth through knowledge transfer and exchange: a research agenda for engaging effectively with policymakers. Reprod Health. 2016;13(1):26. 10.1186/s12978-016-0146-8. 26987438 PMC4797256

[27] Darmstadt GL Bhutta ZA Cousens S Adam T Walker N de Bernis L; Lancet Neonatal Survival Steering Team. Evidence-based, cost-effective interventions: how many newborn babies can we save? Lancet. 2005;365(9463):977–988. 10.1016/S0140-6736(05)71088-6. 15767001

[28] Roberts D Brown J Medley N Dalziel SR. Antenatal corticosteroids for accelerating fetal lung maturation for women at risk of preterm birth. Cochrane Database Syst Rev. 2017;3:CD004454. 10.1002/14651858.CD004454.pub3. 28321847 PMC6464568

[29] Blencowe H Cousens S Mullany LC. Clean birth and postnatal care practices to reduce neonatal deaths from sepsis and tetanus: a systematic review and Delphi estimation of mortality effect. BMC Public Health. 2011;11(suppl 3):S11. 10.1186/1471-2458-11-S3-S11. 21501428 PMC3231884

[30] Allegranzi B Pittet D. Role of hand hygiene in healthcare-associated infection prevention. J Hosp Infect. 2009;73(4):305–315. 10.1016/j.jhin.2009.04.019. 19720430

[31] Arifeen SE Mullany LC Shah R. The effect of cord cleansing with chlorhexidine on neonatal mortality in rural Bangladesh: a community-based, cluster-randomised trial. Lancet. 2012;379(9820):1022–1028. 10.1016/S0140-6736(11)61848-5. 22322124

[32] Mullany LC Darmstadt GL Khatry SK. Topical applications of chlorhexidine to the umbilical cord for prevention of omphalitis and neonatal mortality in southern Nepal: a community-based, cluster-randomised trial. Lancet. 2006;367(9514):910–918. 10.1016/S0140-6736(06)68381-5. 16546539 PMC2367116

[33] Conde-Agudelo A Belizán JM Diaz-Rossello J. Cochrane Review: Kangaroo mother care to reduce morbidity and mortality in low birthweight infants. Evid Based Child Health. 2012;7(2):760–876. 10.1002/ebch.1837 12804436

[34] Silverman WA Fertig JW Berger AP. The influence of the thermal environment upon the survival of newly born premature infants. Pediatrics. 1958;22(5):876–886. 13600915

[35] Kumar V Shearer JC Kumar A Darmstadt GL. Neonatal hypothermia in low resource settings: a review. J Perinatol. 2009;29(6):401–412. 10.1038/jp.2008.233. 19158799

[36] Lunze K Bloom DE Jamison DT Hamer DH. The global burden of neonatal hypothermia: systematic review of a major challenge for newborn survival. BMC Med. 2013;11(1):24. 10.1186/1741-7015-11-24. 23369256 PMC3606398

[37] Nkala T Msuya S. Prevalence and predictors of exclusive breastfeeding among women in Kigoma region, Western Tanzania: a community based cross-sectional study. Int Breastfeed J. 2011;6(1):17. 10.1186/1746-4358-6-17. 22070861 PMC3221641

[38] UNICEF. The State of the World's Children 2008: Child Survival. New York: UNICEF; 2007. https://www.unicef.org/publications/index_42623.html. Accessed April 23, 2019.

[39] Debes AK Kohli A Walker N Edmond K Mullany LC. Time to initiation of breastfeeding and neonatal mortality and morbidity: a systematic review. BMC Public Health. 2013;13(suppl 3):S19. 10.1186/1471-2458-13-S3-S19. 24564770 PMC3847227

[40] Penfold S Hill Z Mrisho M. A large cross-sectional community-based study of newborn care practices in southern Tanzania. PLoS One. 2010;5(12):e15593. 10.1371/journal.pone.0015593. 21203574 PMC3006340

[41] Hill Z Tawiah-Agyemang C Manu A Okyere E Kirkwood BR. Keeping newborns warm: beliefs, practices and potential for behaviour change in rural Ghana. Trop Med Int Health. 2010;15(10):1118–1124. 10.1111/j.1365-3156.2010.02593.x. 20667049

[42] Lee ACC Cousens S Wall SN. Neonatal resuscitation and immediate newborn assessment and stimulation for the prevention of neonatal deaths: a systematic review, meta-analysis and Delphi estimation of mortality effect. BMC Public Health. 2011;11(suppl 3):S12. 10.1186/1471-2458-11-S3-S12. 21501429 PMC3231885

[43] Lawn JE Lee AC Kinney M. Two million intrapartum‐related stillbirths and neonatal deaths: where, why, and what can be done? Int J Gynaecol Obstet 2009;107(suppl 1):S5–S18, S19. 10.1016/j.ijgo.2009.07.016. 19815202

[44] Bang AT Bang RA Baitule SB Reddy HM Deshmukh MD. Management of birth asphyxia in home deliveries in rural Gadchiroli: the effect of two types of birth attendants and of resuscitating with mouth-to-mouth, tube-mask or bag-mask. J Perinatol. 2005;25(suppl 1):S82–S91. 10.1038/sj.jp.7211275. 15791282

[45] Carlo WA Goudar SS Jehan I. High mortality rates for very low birth weight infants in developing countries despite training. Pediatrics 2010;126(5):e1072–e1080. 10.1542/peds.2010-1183. 20937655 PMC3918943

[46] Kamath BD MacGuire ER McClure EM Goldenberg RL Jobe AH. Neonatal mortality from respiratory distress syndrome: lessons for low-resource countries. Pediatrics. 2011;127(6):1139–1146. 10.1542/peds.2010-3212. 21536613 PMC9923778

[47] PATH. Intrapartum-related events rapid landscape analysis. 2012.

[48] Jobe AH Ikegami M. Lung development and function in preterm infants in the surfactant treatment era. Annu Rev Physiol. 2000;62(1):825–846. 10.1146/annurev.physiol.62.1.825. 10845113

[49] Bang AT Bang RA Stoll BJ Baitule SB Reddy HM Deshmukh MD. Is home-based diagnosis and treatment of neonatal sepsis feasible and effective? Seven years of intervention in the Gadchiroli field trial (1996 to 2003). J Perinatol. 2005;25(suppl 1):S62–S71. 10.1038/sj.jp.7211273. 15791280

[50] Ganatra HA Zaidi AK. Neonatal infections in the developing world. Semin Perinatol. 2010;34(6):416–425. 10.1053/j.semperi.2010.09.004. 21094416

[51] McPheeters ML Miller WC Hartmann KE. The epidemiology of threatened preterm labor: a prospective cohort study. Am J Obstet Gynecol. 2005;192(4):1325–1329, discussion 1329–1330. 10.1016/j.ajog.2004.12.055. 15846230

[52] Gazmararian JA Petersen R Jamieson DJ. Hospitalizations during pregnancy among managed care enrollees. Obstet Gynecol. 2002;100(1):94–100. 12100809 10.1016/s0029-7844(02)02024-0

[53] Scott C Chavez G Atrash H Taylor D Shah R Rowley D. Hospitalizations for severe complications of pregnancy, 1987–1992. Obstet Gynecol. 1997;90(2):225–229. 10.1016/S0029-7844(97)00230-5. 9241298

[54] Kamath-Rayne BD MacGuire ER McClure EM Goldenberg RL Jobe AH. Clinical algorithms for the identification of sick newborns in community-based settings. Acta Paediatr. 2012;101(4):344–351. 10.1111/j.1651-2227.2011.02540.x. 22122011

[55] Yee WH Soraisham AS Shah VS Aziz K Yoon W Lee SK; Canadian Neonatal Network. Incidence and timing of presentation of necrotizing enterocolitis in preterm infants. Pediatrics. 2012;129(2):e298–e304. 10.1542/peds.2011-2022. 22271701

[56] Uauy R Fanaroff A Korones S.; National Institute of Child Health and Human Development Neonatal Research Network. Necrotizing enterocolitis in very low birth weight infants: biodemographic and clinical correlates. J Pediatr. 1991;119(4):630–638. 10.1016/S0022-3476(05)82418-7. 1919897

[57] Khanal S Sharma J Gc VS. Community health workers can identify and manage possible infections in neonates and young infants: MINI—a model from Nepal. J Health Popul Nutr. 2011;29(3):255–264. 10.3329/jhpn.v29i3.7873. 21766561 PMC3131126

[58] Van den Bruel A Haj-Hassan T Thompson M Buntinx F Mant D; European Research Network on Recognising Serious Infection investigators. Diagnostic value of clinical features at presentation to identify serious infection in children in developed countries: a systematic review. Lancet. 2010;375(9717):834–845. 10.1016/S0140-6736(09)62000-6. 20132979

[59] United Nations Children's Fund (UNICEF); World Health Organization (WHO). Low Birthweight: Country, Regional and Global Estimates. New York and Geneva: UNICEF and WHO; 2004. https://www.unicef.org/publications/files/low_birthweight_from_EY.pdf. Accessed April 23, 2019.

